# Skin miRNA profiling reveals differentially expressed miRNA signatures from non-segmental vitiligo patients

**DOI:** 10.1186/1755-8166-7-S1-P118

**Published:** 2014-01-21

**Authors:** Mohmmad Shoab Mansuri, Mala Singh, Naresh C Laddha, Mitesh Dwivedi, Yogesh S Marfatia, Rasheedunnisa Begum

**Affiliations:** 1 Department of Biochemistry, Faculty of Science, The M.S. University of Baroda, Vadodara, Gujarat, India-390002; 2 Department of Skin and V.D., Faculty of Medicine, The M.S. University of Baroda, Vadodara, Gujarat, India-390002

## 

miRNAs are small conserved non-coding RNA molecules that post-transcriptionally regulate gene expression by targeting the 3’ UTR of specific mRNA for degradation or translational repression. miRNAs have been shown to be promising biomarkers for different diseases. At present, the expression and function of miRNAs in human skin is largely unknown. The aim of the present study was to detect the differentially expressed miRNAs in non-segmental vitiligo (NSV) patients and to explore the potential role of these miRNAs in vitiligo pathogenesis. We performed the whole miRNA profiling of lesional as well as non-lesional skin from four NSV patients and four healthy skin samples from controls using TaqMan^®^ Low Density Array. Our results suggest that 38 miRNAs were differentially expressed in the skin of patients compared to controls. We identified 13 miRNAs which were significantly differentially expressed in lesional skin of patients compared to healthy control skin. Further, 29 miRNAs were found to be significantly differentially expressed between non-lesional skin of patients and healthy control skin. Interestingly, three miRNAs were specifically down-regulated in the lesional skin compared to non-lesional skin from patients with NSV. In conclusion, for the first time the present study suggests the crucial role of differentially expressed miRNAs in NSV patients from Gujarat.

